# Targeted Delivery Strategies for Multiple Myeloma and Their Adverse Drug Reactions

**DOI:** 10.3390/ph17070832

**Published:** 2024-06-25

**Authors:** Shuting Li, Hongjie Wang, Shijun Xiong, Jing Liu, Shuming Sun

**Affiliations:** 1 Xiangya School of Medicine, Central South University, Changsha 410011, China; 8303201517@csu.edu.cn (S.L.); 8303201304@csu.edu.cn (H.W.); xsj1999411@163.com (S.X.); jingliucsu@hotmail.com (J.L.); 2 Department of Biochemistry and Molecular Biology, Center for Medical Genetics, School of Life Sciences, Hunan Province Key Laboratory of Basic and Applied Hematology, Central South University, Changsha 410011, China

**Keywords:** multiple myeloma, drug delivery system, targeted therapy

## Abstract

Currently, multiple myeloma (MM) is a prevalent hematopoietic system malignancy, known for its insidious onset and unfavorable prognosis. Recently developed chemotherapy drugs for MM have exhibited promising therapeutic outcomes. Nevertheless, to overcome the shortcomings of traditional clinical drug treatment, such as off-target effects, multiple drug resistance, and systemic toxicity, targeted drug delivery systems are optimizing the conventional pharmaceuticals for precise delivery to designated sites at controlled rates, striving for maximal efficacy and safety, presenting a promising approach for MM treatment. This review will delve into the outstanding performance of antibody–drug conjugates, peptide–drug conjugates, aptamer–drug conjugates, and nanocarrier drug delivery systems in preclinical studies or clinical trials for MM and monitor their adverse reactions during treatment.

## 1. Introduction

Multiple myeloma (MM), also known as plasma cell myeloma, is a hematologic system cancer characterized by the uncontrollable growth of plasma cells. It constitutes roughly 10% of all hematologic malignancies [1]. Most commonly in MM cases, the production of monoclonal immunoglobulins results in organ damage, clinically presenting as the classic combination of elevated blood calcium, renal impairment, anemia, and bone pain. Additionally, patients may experience sensory abnormalities, hepatosplenomegaly, lymphadenopathy, and fever. Clinical staging, employing various methods, categorizes the disease into stages I, II, and III based on its severity.

Epidemiological studies demonstrate that between 1990 and 2019, the global incidence and mortality of MM more than doubled over 30 years. In 2019, there were 155,688 reported cases of MM (95% uncertainty interval: 136,585–172,577), with approximately 54.3% of these cases affecting males [2]. MM follows an age-related incidence pattern [3], with a median onset age of 69 years, and approximately 63% of patients are aged 65 and older at the time of diagnosis [4]. Although contemporary treatment approaches have significantly prolonged the median survival time for MM to 128 months [5], a considerable improvement from the 30 months observed before 2000, the disease frequently manifests with an insidious onset, leading to a high rate of misdiagnoses and late-stage detection. This, in turn, results in substantial prognostic variations and has a significant impact on the quality of life of affected individuals [6].

Emerging therapeutic approaches for MM encompass the following: (1) Proteasome inhibitors: These agents facilitate programmed cell death by promoting the buildup of aberrant and ubiquitinated proteins within cells and preventing the breakdown of pro-apoptotic factors. Bortezomib (BTZ), a well-established drug, represents a standard treatment option for MM, while the subsequently developed carfilzomib (CFZ) has demonstrated comparable or even superior efficacy. (2) Immunomodulators: Immunomodulators can bind to the E3 ubiquitin ligase cereblon, triggering proteasome-dependent degradation of Ikaros family zinc finger proteins 1 and 3, thereby facilitating immunomodulators’ cytotoxic and immune-modulatory effects in MM cells. Moreover, their mechanism also involves stimulation of natural killer cell activity, inhibition of regulatory T cells, prevention of MM cell adhesion to bone marrow (BM) stromal cells and caspase-8-mediated apoptosis. Representative drugs in this category include thalidomide, lenalidomide, and pomalidomide. (3) Histone deacetylase inhibitors: Inhibitors like panobinostat, ricolinostat, and vorinostat target histone deacetylase 6, inhibiting proteasome formation and the degradation of misfolded proteins, ultimately promoting cell apoptosis. (4) Monoclonal antibodies (mAbs): Anti-CD38 monoclonal antibodies, such as daratumumab, isatuximab, target overexpressed CD38 on MM cells, block cell adhesion and proliferation, and induce cell apoptosis. (5) Bispecific antibodies: They can simultaneously target antigens on malignant plasma cells and cytotoxic immune effector cells, making them a critical immunotherapy. For example, teclistamab induces cytotoxicity against MM cells by binding to both the B-cell maturation antigen (BCMA) and CD3 antigens, stimulating T cells to release cytotoxins. Additionally, ongoing advancements in MM treatment include chimeric antigen receptor (CAR)-T cell therapy and immune checkpoint inhibitors. Despite the substantial efficacy and betterment in patient-reported outcomes associated with proteasome inhibitors and immunomodulator therapies, these treatments lack precise lesion targeting and can result in off-target adverse effects. Furthermore, MM cells are developing multidrug resistance to agents like BTZ and thalidomide, diminishing treatment effectiveness. Hence, new drug delivery systems that precisely target tumors urgently need to be developed [7,8,9,10,11]. 

Drug delivery systems (DDSs) constitute an interdisciplinary field spanning medicine, engineering, and pharmaceuticals. DDS encompasses the study of drugs and their carriers, including physicochemical modifications, to attain precise drug delivery at the correct location and moment. After years of development, DDS now encompasses diverse systems, such as antibody–drug conjugates (ADCs), peptide–drug conjugates, aptamer–drug conjugates, and nanomedicines. 

DDS offers numerous advantages and holds promising prospects. In the contemporary landscape of costly and time-consuming new drug development, emerging targeted drug conjugates and nanomedicines optimize and enhance existing medications. They deliver drugs precisely to designated sites at predetermined rates to maximize efficacy and safety—a concept often referred to as ‘the last mile’ of drug development. Leveraging the well-established efficacy of existing drugs, emerging DDSs provide comprehensive control over drug distribution in terms of timing, location, and dosage. This approach offers enhanced selectivity, mitigates drug resistance, reduces systemic toxicity, improves safety profiles, regulates drug release kinetics, minimizes plasma drug level fluctuations, boosts delivery efficiency, and holds potential economic benefits. Targeted DDSs have made significant strides at both macroscopic and nanoscale levels, establishing themselves as a pivotal strategy in cancer treatment [12].

**Figure 1:**
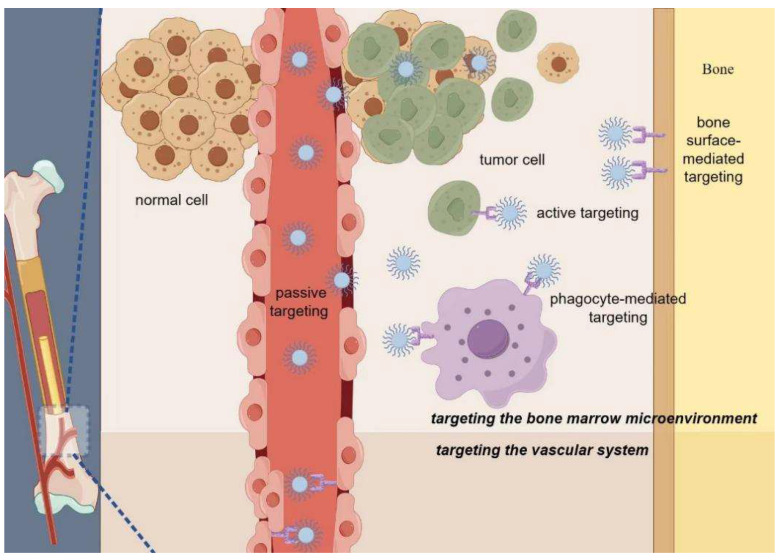
Drug targeting delivery strategies for multiple myeloma (By Figdraw).

Overall, the existing DDS for MM primarily includes two approaches: targeting the vascular system and targeting the BM microenvironment [13]. The abundant neovascularization in the BM of MM patients, linked to an unfavorable prognosis, provides the foundation for focusing on the vascular system in the treatment of MM [14]. Vascular-targeted DDS delivers anti-angiogenic agents to inhibit angiogenesis and release chemotherapy drugs, suppressing cell proliferation near BM vasculature and enhancing MM therapy [13]. Strategies for targeting the BM and microenvironment can be categorized into passive targeting, bone-surface-mediated targeting, phagocyte-mediated targeting, and active targeting. Passive targeting involves DDSs that evade the body’s elimination mechanisms and accumulate at specific disease sites through leaky blood vessels [15]. By precisely modulating the delivery system’s size, shape, structure, and surface properties, it can penetrate the BM through endothelial intercellular and intracellular pathways, independently of ligand recognition. Bone-surface-mediated targeting relies mainly on substances with a high affinity for hydroxyapatite in the bone. Various peptides formed by glutamic acid or aspartic acid [16] and bisphosphonates [14] have been shown to have the potential for drug-targeted delivery to the BM. Phagocyte-mediated targeting is based on the characteristics of certain macrophages entering the BM and is considered a possible opportunity for drug delivery. Macrophages express several surface receptors, such as mannose receptors and galactose receptors [15], which can be used to find specific ligands for drug-targeted delivery. However, because almost all macrophages express the same receptors, this strategy is limited in practical application. Active targeting enhances specific drug delivery [13]. MM cells exhibit distinct cell surface biomarkers that enable selective binding to peptides, antibodies, aptamers, and other ligands on the delivery system’s surface, thus enabling precise drug delivery. This active targeting enhances the specificity of drug delivery to the tumor microenvironment and tissues (Figure 1).

## 2. Antibody–Drug Conjugates

ADCs link antibodies to traditional or novel chemotherapeutic agents, directing them to specific tumor cell surface antigens. This precision in drug delivery minimizes off-target effects and adverse reactions, leading to improved clinical outcomes. Although the concept of ADCs dates back to the 1960s, it has evolved through three generations of drug development, enhancing their efficacy and stability [17]. Today, ADCs are integral to the targeted treatment of malignancies, including MM. The FDA has sanctioned Belantamab mafodotin as the inaugural ADC for MM therapy [18], demonstrating significant anti-myeloma efficacy and a controllable safety profile in patients with relapsed or refractory MM. Moreover, numerous ADCs aimed at MM cell surface antigens are currently under clinical evaluation.

### 2.1. Structure and Mechanism of ADCs

**Figure 2:**
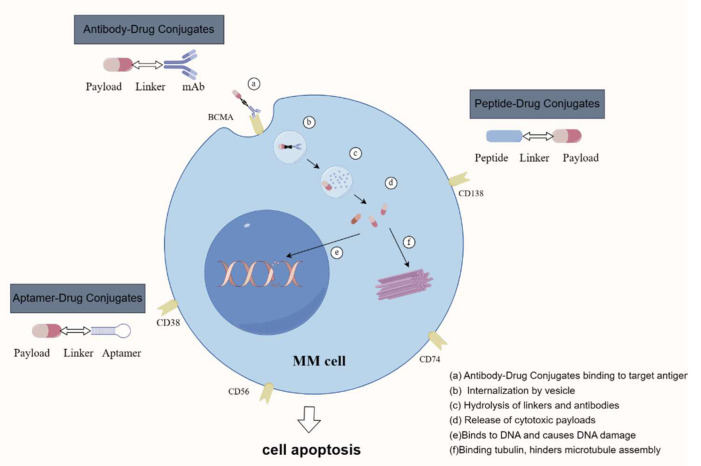
Structure and mechanism of ligand–drug conjugates (By Figdraw).

ADCs consist of three primary elements: tumor-specific mAbs, cytotoxic small molecules forming the active payload, and a bespoke chemical linker that attaches the mAb to the payload [19]. In an ideal scenario, the mAb-targeted antigens are predominantly expressed in tumors, scarcely or not at all in normal tissues, and exhibit robust internalization and uptake, enabling the rapid and specific delivery of cytotoxic drugs into tumor cells. ADCs commonly target MM cell surface antigens such as BCMA, CD38, CD138, and FcRH5. The payloads in ADCs are usually traditional cytotoxic drugs, divided into two classes: microtubule inhibitors and DNA damaging agents [17]. Despite the notable efficacy of frontline treatments like BTZ, lenalidomide, and daratumumab, their application in ADCs remains limited. Chemical linkers in ADCs are mainly composed of cleavable (acid-labile, protease-cleavable, and disulfide bond linkers) and non-cleavable types. The cleavable linkers are designed to break down under specific cellular conditions, whereas the non-cleavable linkers are more robust, relying on lysosomal degradation of the mAb framework to release the active payload [20,21,22]. These linkers are engineered to attach to designated amino acid residues on the monoclonal antibody, while the opposite end forms a chemical bond with the small molecule drug, ensuring stability during circulation and effective release upon target cell entry. In the systemic administration of ADCs for relapsed or refractory MM patients, the monoclonal antibody initially targets and binds to antigens on the MM cell surface, facilitating ADC internalization. Subsequently, the linker undergoes cleavage in the unique cellular milieu, liberating the cytotoxic drug that induces cellular damage and apoptosis (Figure 2).

### 2.2. ADCs for MM Treatment

The tumor necrosis factor receptor superfamily 17’s BCMA shows high expression on malignant plasma cells and late B lymphocytes’ surfaces. On hematopoietic stem cells, however, it exhibits little expression and is missing on non-hematopoietic cells [23,24]. BCMA possesses two activating ligands: a proliferation-inducing ligand and B-cell activating factor, predominantly secreted in a paracrine fashion by BM stromal cells, osteoclasts, and macrophages located within the BM. Consequently, BCMA activation triggers numerous proliferative and anti-apoptotic signaling cascades in MM cells. These pathways include the NF-κB signaling route, leading to upregulation of anti-apoptotic proteins, as well as the generation of cell adhesion molecules, factors promoting angiogenesis, and molecules with immunosuppressive properties, ultimately fostering the heightened survival of MM cells [25]. Hence, BCMA serves as a therapeutic target within the realm of DDS tailored for MM. Researchers have leveraged humanized antibodies targeting BCMA to design numerous highly efficient ADC delivery systems.

**Belantamab mafodotin (belamaf)** is an IgG1 mAb targeting BCMA, engineered with a afucosylated Fc region (J6MO). It is connected to a maleimide-caproyl linker that cannot be cleaved, along with monomethyl auristatin F (MMAF, mafodotin) [26]. Preclinical data demonstrate that belamaf induces apoptosis in malignant plasma cells through four primary mechanisms [27]. (1) J6MO recognizes the cell surface antigen BCMA on MM cells, undergoes internalization, and subsequently undergoes complete lysosomal degradation, liberating the cytotoxic drug mafodotin. Attaching to tubulin, mafodotin obstructs microtubule assembly, leading to cell cycle arrest at G2/M and apoptosis that relies on Caspase-3. (2) Afucosylated functionalized J6MO antibodies recruit effector cells like NK cells, augmenting antibody-dependent cell-mediated cytotoxicity. (3) In addition to its primary mechanisms, belamaf can initiate immunogenic cell death, causing apoptotic cancer cells to release neoantigens that activate immune effector cells, promote dendritic cell maturation, and stimulate macrophage-mediated antibody-dependent cell phagocytosis against tumor cells. (4) Naked antibody J6MO can block signaling by the B-cell activating factor and a proliferation-inducing ligand through BCMA, resulting in the demise of plasma cells. Belantamab mafodotin has demonstrated notable efficacy in the DREAMM-2 clinical trial. The most common Grade 3–4 adverse events were corneal disorders (26 out of 95 patients [27%] in the 2.5 mg/kg group, 21 out of 99 patients [21%] in the 3.4 mg/kg group), thrombocytopenia (19 pts [20%] and 33 pts [33%]), and anemia (19 pts [20%] and 25 pts [25%]) [28]. It received its initial approval in the United States on 5 August 2020, for adult patients with MM who had relapsed or were refractory and who had received a minimum of four previous treatments [18]. Despite belamaf exhibiting substantial single-agent efficacy, it failed to achieve the primary endpoint in the phase III clinical trial DREAMM-3. The median progression-free survival in the belamaf monotherapy group was statistically indistinguishable from the pomalidomide-dexamethasone combination group (hazard ratio 1.03 [0.72–1.47]; *p* = 0.56). Meanwhile, DREAMM-3 revealed that the most common Grade 3–4 adverse events in the monotherapy group treated with belamaf were thrombocytopenia (49 out of 217 patients [23%]) and anemia (35 patients [16%]) [29]. Owing to the absence of novel serious adverse reactions, belamaf remains in use within combination therapy protocols for relapsed or refractory MM, aiming to assess its synergistic effects with other active anti-MM agents. DREAMM-7 is a phase 3, open-label, randomized trial that evaluated the efficacy of combining belantamab mafodotin, BTZ, and dexamethasone (BVd) versus the combination of daratumumab, BTZ, and dexamethasone (DVd) in patients with refractory MM [30]. The PFS in the BVd group was significantly higher than in the DVd group, at 36.6 months (95% CI, 28.4 to not reached) versus 13.4 months (95% CI, 11.1 to 17.5). The overall survival rate at 18 months was 84% for the BVd group compared to 73% for the DVd group. This indicates that BVd has greater benefits in terms of PFS as well as deeper and more sustained responses compared to DVd. In the BVd group, ocular events remained the most significant adverse reaction, occurring more frequently (79% vs. 29%) than in the DVd group, but most visual impairment events could be resolved through dose adjustment. Concurrently, the phase 3 clinical trial DREAMM-8 demonstrated that the combination of belantamab mafodotin, pomalidomide, and BTZ has significant benefits in terms of PFS [31]. Therefore, the combination of belamaf with other drugs for treating MM shows excellent clinical efficacy and is expected to become a new treatment strategy.

Furthermore, a multitude of ADCs delivery systems have been engineered with BCMA as the target. **HDP-101**, a fully human mAb directed at BCMA, is covalently bonded to an amanitin derivative via an indissoluble MC linker [32]. α-Amanitin, an octapeptide with a bicyclic structure, functions as an inhibitor of eukaryotic RNA polymerase II, capable of hindering cellular transcription and protein synthesis, even in trace amounts, thereby inducing apoptosis [33]. Demonstrated through cell-based and animal studies, HDP-101 has shown considerable effectiveness against both proliferative and dormant MM cells. Additionally, administration to cynomolgus monkeys at various dosages revealed no significant hepatic or renal toxicity, suggesting favorable tolerability and a hopeful therapeutic index [32]. A phase 1/2a first-in-human trial is currently in progress, aiming to assess HDP-101’s safety, tolerability, pharmacodynamics, and therapeutic efficacy in MM patients [34]. As of July 2023, in the initial human trials, the first three cohorts were well tolerated with no dose-limiting toxicities, no signs of hepatic or renal toxicity, no infusion reactions detected, and no reports of corneal disorders or vision impairment, indicating a promising therapeutic outlook. However, the ADC carrier drug amanitin has strong hepatic and renal toxicity, so close monitoring of adverse reactions of the drug in humans is still required.

**MEDI2228** conjugates a pyrrolobenzodiazepine dimer, a potent DNA minor groove cross-linking agent, to a humanized antibody via a protease-cleavable linker [25]. Initial human trials revealed significant adverse reactions, treatment-related adverse events occurring in ≥20% of patients were photophobia (53.7%), thrombocytopenia (31.7%), rash (29.3%), elevated gamma-glutamyl transferase (24.4%), dry eye (19.5%), and pleural effusion (19.5%), casting doubt on the monotherapy’s safety profile [35]. However, co-administration with other pharmaceuticals may mitigate MEDI2228’s toxicity and enhance therapeutic outcomes. Research by Xing et al. demonstrated that the combination of MEDI2228 with BTZ augments irreversible DNA damage and tumor cell mortality, yielding an improved therapeutic response compared to monotherapy [36]. Furthermore, MEDI2228 was found to elevate CD38 expression on MM cells through an INF-driven immune response, counteracting the CD38 downregulation or absence in daratumumab-resistant MM cells, thus potentiating daratumumab’s efficacy [37]. This evidence strongly supports the combined use of MEDI2228 and daratumumab to enhance patient outcomes. These insights advocate for the integration of MEDI2228 with BTZ or daratumumab as a novel combinatory approach, bolstering its clinical development for relapsed and refractory MM patients. ADCs targeting other cell surface markers on MM cells are listed in the table below (Table 1).

## 3. Peptide–Drug Conjugates

Peptide–drug conjugates (PDCs) represent a category of targeted therapeutic agents akin in structure to ADCs, differing in the use of distinct ligands for targeting. PDCs comprise three essential elements: targeting peptide, conjugation linker, and cytotoxic warhead [46]. These components collaborate synergistically to target specific receptors on tumor cells, facilitating the delivery of cytotoxins.

Melflufen, a novel peptide–drug conjugate for relapsed/refractory MM, links melphalan—a well-established anti-MM agent—with para-fluoro-L-phenylalanine ethyl ester via a peptide bond. It garnered its inaugural approval in the United States on 26 February 2021 [47]. Melflufen enhances intracellular melphalan levels at reduced concentrations more rapidly than melphalan alone, significantly decreasing the half-maximal inhibitory concentration. Additionally, melflufen prompts apoptosis in melphalan-resistant MM cells [48], suggesting an alternative tumoricidal mechanism. Preclinical research has elucidated that melflufen eradicates tumor cells predominantly through the following mechanisms: (a) Melflufen, with its high lipophilicity, traverses cell membranes by passive diffusion and undergoes hydrolysis by intracellular aminopeptidase, liberating substantial quantities of melphalan, which, due to its lower lipophilicity, is retained within the cell for extended periods. This process significantly elevates the intracellular concentration of melphalan, triggering DNA damage and apoptosis via a p53-independent pathway [49]. (b) Melflufen activates the unfolded protein response through the protein kinase RNA-like endoplasmic reticulum kinase pathway, enhancing the expression of C/EBP homologous protein and consequently inducing apoptosis [50]. (c) Melflufen interferes with the differentiation of human BM-derived mesenchymal stem cells into adipocytes and osteoblasts, as well as BMSC-mediated angiogenesis, thereby disrupting the BM microenvironment and attenuating the BM-derived mesenchymal stem cells’ supportive function for myeloma cells [51].

**O-12-M1**, a multicenter, open-label phase 1/2 clinical trial, evaluated the safety and efficacy of melflufen in combination with dexamethasone. Phase 1 established the maximum tolerated dose of melflufen as 40 mg, with the most common adverse reactions being clinically manageable thrombocytopenia, neutropenia, and pneumonia. Phase 2 demonstrated that the combination therapy of melflufen plus dexamethasone was more effective than melflufen monotherapy, with overall response rates of 31% (95% CI 18–47) and 8% (0.2–36.0), respectively [52]. The phase II **HORIZON** trial enrolled 157 patients to further investigate the effects of the combination therapy of melflufen (40 mg) with dexamethasone. The results indicated that among the entire treatment cohort, the overall response rate was 29%, with a median duration of response of 5.5 months, median progression-free survival of 4.2 months, median overall survival of 11.6 months, and a median follow-up time of 14 months, with adverse reactions similar to previous findings [49]. The significant clinical efficacy and reliable safety profile of HORIZON led to the FDA’s accelerated approval of the combination of melflufen with dexamethasone for the treatment of adults with relapsed or refractory MM. In the phase III **OCEAN** trial, melflufen in combination with dexamethasone did not exhibit clinical advantages over pomalidomide plus dexamethasone, with melflufen’s median overall survival reaching 19.8 months, which was shorter than the latter’s 25.0 months [53]. The FDA, citing the trial’s negative impact on overall survival, halted enrollment and suspended other ongoing trials such as ANCHOR, BRIDGE, PORT, and LIGHTHOUSE. On 23 February 2024, the FDA withdrew melflufen’s approval due to insufficient evidence of clinical benefit. Despite the study’s early termination affecting patient numbers and follow-up duration, the final outcomes of the **ANCHOR** and **LIGHTHOUSE** studies revealed promising, durable clinical responses from the triple therapy of melflufen, daratumumab, and dexamethasone, endorsing its real-world application [54,55]. Future research may explore melflufen’s potential in combination therapies for relapsed or refractory MM.

## 4. Aptamer–Drug Conjugates

### 4.1. Introduction to Aptamers and APDC Systems

Nucleic acid aptamers, colloquially termed ‘chemical antibodies’, are concise single-stranded DNA or RNA sequences that bind selectively to a variety of targets, ranging from small metal ions and organic compounds to proteins, entire cells, and even living animal tissues and organs [56]. These aptamers adopt distinctive three-dimensional configurations, enabling high-affinity and specific interactions with their targets through electrostatic forces, hydrogen bonding, and van der Waals interactions [57]. Functionally analogous to protein antibodies, aptamers possess several superior attributes: (i) small size with higher tissue penetration; (ii) extremely low immunogenicity; (iii) easy conjugation with various functional agents; and (iv) low cost and fast chemical synthesis.

Aptamers can independently serve as therapeutic drugs for cancer-targeted therapy, with the main mechanism being blocking interactions of disease-related targets. Currently, many aptamer drugs have entered clinical trial stages, such as the VEGF-targeting aptamer pegaptanib for ophthalmic diseases [58]. Moreover, numerous aptamers can enter cells by binding to cell surface receptors, allowing them to serve as effective targeting moieties for conventional drugs, oligonucleotide drugs, etc. Similar to ADCs, ApDCs typically consist of three parts: the aptamer, the linker, and the drug. The nucleic acid aptamer serves as the targeting ligand, guiding the delivery of the therapeutic drug to the targeted lesion and regulating the biological function of the target biomarker. A broad spectrum of drugs can be conjugated to aptamers, including traditional chemotherapeutics, oligonucleotide drugs (siRNAs, shRNAs, miRNAs, anti-miRs), radiopharmaceuticals, photosensitizers, etc.

### 4.2. Aptamer Delivery Systems in MM

Based on different cell surfaces or intracellular biomarkers of MM cells, specific aptamers can be chemically conjugated to MM therapeutic drugs to design APDCs targeting different sites. Previous studies have proven the aptamer–miRNA conjugates apt69.T-miRNA-137 and apt69.T-anti-miR-222 targeting BCMA and the aptamer–drug conjugate #1S-DOX targeting CD38 significantly inhibit MM cell activity, showing promise as emerging drugs to treat MM.

**APDCs Targeting BCMA** Silvia Catuogno and her colleagues initially synthesized an RNA aptamer called apt69.T [59], which exhibits a specific affinity for BCMA. Apt69.T is modified with 2’Fluoro-Pyrimidine to enhance its resistance to nuclease degradation and has strong serum stability. Apt69.T can reduce a proliferation-inducing ligand-induced p65 phosphorylation and downstream effector extracellular signal-regulated kinase 1/2 phosphorylation. In addition to serving as a therapeutic itself, it can also be used as a carrier to deliver oligonucleotide drugs. In subsequent studies, two distinct conjugates were designed. The first conjugate conjugated the non-coding strand of human miRNA-137 to the 3′ end of apt69.T. miR-137 is reportedly a tumor-suppressive miRNA in MM that is present at very low concentrations intrinsically. The apt69.T-137 conjugate induced a significant increase in the expression of miR-137 in U266 cells that had BCMA receptors, and U266 cell viability was significantly decreased, while only a slightly elevated miR-137 level was observed in BCMA-negative CCRF-CEM cells, with no significant change in cell viability. The second conjugate linked apt69.T to the single-stranded molecule that inhibits miR-222, which is an oncogenic microRNA that has been discovered to be abnormally highly expressed in MM cells. The T-anti-miR-222 conjugate significantly downregulated miR-222 expression, producing a therapeutic effect. In summary, the findings suggest that apt69.T has the potential to function as an exceptionally effective and specific carrier for therapeutic oligonucleotides. This portrays a promising avenue for targeted therapy in MM and offers both a conceptual foundation and evidence for the development of innovative and efficient aptamer-based targeted treatments for MM patients.

**APDCs Targeting CD38** ADCs targeting CD38 have been developed, combining targeted antibodies with cytotoxic drugs, exhibiting potent anti-tumor activity. However, the manufacturing of ADCs is costly, time-consuming, and labor-intensive, and aptamers happen to overcome this issue. After multiple rounds of SELEX screening and structural optimization, J Wen et al. prepared an aptamer #1S with the highest binding affinity and specificity for CD38. Both in vitro and in vivo experiments demonstrated aptamer #1S specifically targets MM tumors and selectively accumulates [60]. In follow-up studies, a CG-cargo was conjugated to the 3′/5′ ends of aptamer #1S, carrying a substantial amount of doxorubicin (DOX) molecules via non-covalent intercalation, with each #1S aptamer fully binding 5 DOX payloads at a 1:5 molar ratio of the aptamer to DOX to form an APDC. The administration of ApDCs systemically leads to their specific targeting of CD38 present in myeloma cells, resulting in receptor-mediated internalization into the malignant cells. Upon being internalized into myeloma cells, ApDCs undergo transport to lysosomes, where the acidic microenvironment with a pH around 5 induces conformational alterations in ApDCs, enabling rapid intracellular discharge of doxorubicin payloads carried within ApDCs. This rapid unloading of doxorubicin inside myeloma cells triggers their death. Notably, APDC treatment had a more significant apoptotic effect than free DOX in DOX-resistant multidrug-resistant RPMI-Dox40 cells. Additionally, in MM tumor xenografts, comparable doses of APDC gave significantly greater tumor inhibition compared to free DOX, prolonging survival in MM-tumor-bearing mice without causing detectable adverse reactions. To examine the in vivo activity and toxic side effects of the #1S-DOX conjugate, further improved experiments and preclinical testing are still needed.

Many other aptamers targeting surface markers on MM cells also have potential as delivery agents. Activation of the **hepatocyte growth factor/c-met signaling cascade** is involved in the pathogenesis of MM. Therefore, inhibiting this pathway is thought to be a potentially viable therapeutic approach for treating MM. SL1 represents the initial DNA aptamer with specificity for c-met and exhibits promising utility for the targeting and management of MM. SL1 can function both as a molecular probe that binds with high affinity to cells overexpressing c-met in cell cultures and whole organisms and as a therapeutic antagonist for MM cells by inhibiting hepatocyte growth factor-induced c-met signaling. Additionally, SL1 also demonstrates synergistic effects with BTZ. Although SL1 has not yet been utilized to carry drugs for targeted therapy in previous studies, its high affinity and specific binding characteristics suggest it is a highly promising ligand in APDCs, capable of guiding targeted delivery of cytotoxic drugs, oligonucleotide drugs, etc. to specific sites [61]. **Annexin A2,** alternatively recognized as Annexin II, belongs to the superfamily of phospholipid-binding proteins that are mediated by calcium ions. Annexin II is expressed at significantly higher levels on the cytoplasmic membrane of myeloma patient cells compared to healthy cells and is negatively correlated with disease-free and overall survival [62]. There have been reports indicating that Annexin A2 contributes to the adhesion and proliferation of MM within the BM microenvironment [63]. Decreasing the levels of Annexin A2 has been found to suppress MM cell growth and promote apoptosis. Zhou et al. [64] employed protein SELEX technology to screen for an aptamer known as wh6, which targets Annexin II. This aptamer can target MM cells in vitro and in vivo, inhibiting MM cell adhesion and proliferation. So, wh6 is a promising candidate tool that could be used to prepare APDCs.

## 5. Nanoparticle Drug Delivery Systems

**Figure 3:**
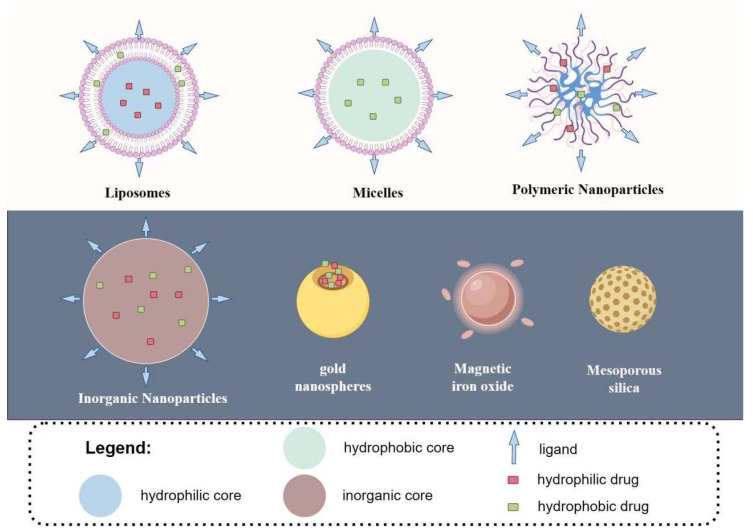
Nanoparticle drug delivery systems (By Figdraw).

In recent years, the development of nanomedicine has flourished and has become an important approach for treating various malignant tumors. The antibodies, peptides, and aptamers mentioned above, as well as molecules like folate and hyaluronic acid, can all serve as ligands for nanocarrier systems, exerting the same targeting effect. The difference lies in the physical encapsulation of drugs by nanocarriers without the need for precise chemical conjugation processes [12]. By loading drugs onto nanoparticles and connecting ligands, precise targeting functions can be achieved. The design and preparation of nanodrug delivery systems with various functions or targeting drugs (genes) can enable molecular diagnostics, drug delivery carriers, and in vivo gene editing [65]. In order to encapsulate and deliver anticancer drugs with different properties, existing nanosystems include organic nanoparticles such as liposomes, micelles, polymer nanoparticles, and various inorganic nanoparticles such as metals and mesoporous silica (Figure 3). The majority of the current research on nanoparticle delivery systems for MM employs a passive targeting strategy [66]. Active targeting can utilize the same drug delivery system as passive targeting but involves attaching ligands to the passive targeting particles to facilitate the delivery of NPs to the destinations.

### 5.1. Liposomes

Liposomes are spherical vesicles consisting of concentric phospholipid bilayers surrounding an aqueous core, with diameters ranging from 20 nm to 1000 nm [67]. Hydrophilic drugs are enclosed within the aqueous core of liposomes, whereas hydrophobic drugs can be trapped within the hydrocarbon chains of the lipid bilayers, rendering liposomes a versatile drug delivery system. Since the approval of Doxil, the first nanoscale liposomal drug for clinical use in 1995, liposomes have been extensively employed owing to their biocompatibility, non-immunogenicity, and capacity to improve the water solubility of chemotherapeutic agents. Furthermore, research has revealed that MM is characterized by heightened microvascular density [68,69] and enlarged endothelial cell fenestrations [66], resulting in enhanced permeability that facilitates the passive targeting of liposomal delivery systems.

Liposomes modified with methoxypolyethylene glycol, also called PEGylated long-circulating liposomes, are crucial carriers. Doxil, a potent cardiotoxic agent, can only mitigate side effects by reducing dosage, which compromises its efficacy [70]. Encapsulation of PEGylated liposomes enables evasion of clearance by the mononuclear phagocyte system. Their prolonged circulation effectively enhances drug concentration in the bloodstream and tumor sites, facilitating passive targeting while minimizing toxic side effects resulting from accumulation non-tumor tissues including cardiac [71]. Recent in vitro studies utilizing PEGylated long-circulating liposomes encapsulating homoharringtonine [72] and dexamethasone [73] have demonstrated significant inhibition of MM cancer stem cells. Specifically, clinical trials involving pegylated liposomal dexamethasone phosphate infusion in relapsed or progressive MM patients have revealed higher drug concentration, prolonged duration, and favorable patient tolerance without additional adverse events compared to traditional drugs. Nonetheless, this study encompassed only seven male cases, requiring larger sample clinical trials and evaluations of the impact of drug administration methods on outcomes to better apply it in MM treatment [74].

Utilizing natural lipid bilayer nanocarriers named apoptotic vesicles for the natural synthesis of liposomal drugs signifies a noteworthy advancement [75]. Mesenchymal stem cells adeptly encapsulate BTZ within apoptotic vesicles, thereby mitigating problems such as inadequate stability and exorbitant costs linked to conventional loading techniques. Cao, Zeyuan et al. illustrated that the activation of Rab7 can enhance the production efficiency of BTZ-apoptotic vesicles. The data indicate treatment with Rab7 activators increased the average synthesis rate of PC-apoptotic vesicle from 33.1% to 47.6%, while treatment with inhibitors reduced the synthesis rate to 17.4%. Additionally, BTZ-apoptotic vesicle attained a cellular apoptosis rate nearing 80% in experiments and demonstrated superior anti-tumor effects with minimal adverse effects compared to the control group in murine models [76].

To enhance drug specificity, the strategy for targeted therapy in MM based on the aforementioned non-targeted liposomal drug delivery system is as follows:

Omstead et al. pioneered the synthesis of CD38-targeting peptides. They employed liposomes as carriers, incorporating CD38- and CD138-targeting peptides. This drug demonstrated superior drug delivery efficiency and cytotoxicity in both in vitro and in vivo experiments compared to direct drug administration. Specifically, in vitro experiments showed superior enhancement with CD138-targeting compared to CD38-targeting nanoparticles, while opposite results were observed in in vivo experiments. Hence, it is inferred that CD38 not only acts as a crucial target for MM treatment but also holds promise for application in other CD38-overexpressing tumors. Additionally, it reminds us that success in in vitro experiments does not necessarily translate to in vivo efficacy [77].

Immunotherapy integrated with targeted nanodelivery systems represents a pivotal approach in the targeted management of MM. Immunotherapy for MM encounters some significant challenges. CAR-T cell therapy requires extraction from the patient’s body, demanding advanced technological and economic capabilities. Bispecific T-cell engagers have a short half-life of 2 h and inadequate pharmacokinetic properties. Moreover, they mainly target a single antigen on cancer cells, which could facilitate tumor evasion and recurrence [78,79,80]. Bae, Jooeun et al. devised a nanodelivery system employing a ligand specific to BCMA, capable of eliciting cytotoxic CD8+ T lymphocytes against MM. This drug delivery system demonstrates sustained release, provokes memory cytotoxic CD8+ T lymphocytes, and holds the advantage of prolonged efficacy [81]. Alhallak, Kinan et al. formulated lipid-based dual-specificity T cell engagers featuring dual ligands targeting T cells, anti-CD3 mAbs and mAbs against cancer antigens, and the drug boasts a 60 h half-life, which can significantly lessen dosing frequency, consequently enhancing efficacy and patient adherence in clinical settings. They also developed nanoparticles targeting BCMA/CD3, CS1/CD3, and CD38/CD3 individually, as well as nanoparticles targeting all three antigens BCMA/CS1/CD38/CD34 simultaneously. Experimental results demonstrated that the last one exhibited higher affinity and cytotoxicity against MM [80]. The amalgamation of nanodelivery systems with immunotherapy bears vast prospects.

### 5.2. Micelles

Micelles are formed by a hydrophobic core carrying hydrophobic anticancer drugs surrounded by a hydrophobic monolayer of amphiphilic lipids. The diameter of micellar particles ranges from 10 to 100 nm, a size that is not easily recognized and captured by the reticuloendothelial system in the bloodstream. This size allows the carrier system to stably exist in the bloodstream for a long time, achieving passive targeting to the tumor site through permeability and retention effects, thereby enabling drug accumulation in the tumor microenvironment [82].

Chen et al. prepared and screened daratumumab (Dar) with a density of 3.2 for CD38-positive MM and developed a micellar drug delivery system loaded with CFZ named Dar3.2-PMs-CFZ. In vitro, the drug showed six times greater inhibitory effects on MM cells LP-1 and MM.1S compared to the non-targeted PMs-CFZ. In vivo, Dar3.2-PMs-CFZ outperformed PMs-CFZ and free CFZ in inhibiting tumor growth and prolonging median survival time, with minimal toxicity, making it a safe and effective treatment for MM [83].

CD44-targeted drugs are an ideal strategy for MM management. CD44, a glycoprotein, is abundantly expressed on various malignant tumors, including MM [84]. Studies show that CD44-targeted nanomedicines utilize hyaluronic acid, A6 peptide, anti-CD44 antibodies, and aptamers for active targeting [85,86,87]. Researchers have developed hyaluronic-acid-coated, disulfide-crosslinked, boron-loaded biodegradable micelles (HA-CCMs-BP), demonstrating superior efficacy over free BP, achieving a maximum tumor inhibition rate of 81.8% in animal models [88]. HA-CCMs enhance drug accumulation in CD44-overexpressing multiple myeloma due to their small size, stability, and prolonged circulation time [89]. A6, an 8-amino acid peptide, shares homology with CD44 [87,90], activating CD44 adhesion activity, inducing FAK and MEK phosphorylation and inhibiting migration and metastasis of CD44-expressing cells [90]. As a promising ligand for DDS, disulfide-crosslinked A6 peptide-tagged micelles loaded with CFZ hold potential for treating CD44-overexpressing MM [87]. Additionally, Dar3.2-PMs-CFZ targeting CD38(+) MM has shown promising effects in both in vitro and in vivo experiments [83].

### 5.3. Polymeric Nanoparticles

Polymers can form both micelles and polymer nanoparticles (PNPs). The micellization of polymers is based on the spontaneous self-assembly of polymer monomers and thermodynamic factors, while the formation of PNPs is controlled by “kinetic” factors such as temperature, pH, electrolytes, solvent content, etc., through slow rearrangement after the rapid addition of monomers.

CFZ, a second-generation proteasome inhibitor, has been employed broadly in MM therapy. However, it still has limitations in water solubility and biological stability [91,92]. Incorporating CFZ into poly (ethylene glycol)-b-poly(N-2-benzoyloxypropyl methacrylamide) nanoparticles enhances its efficacy against MM cells in vitro and exhibits improved tolerability compared to the approved drug Kyprolis^®^ [93]. Encapsulation of iguratimod (IGU) within poly (lactic-co-glycolic acid) (PLGA) nanoparticles (IGU-PLGA-NPs) triggers apoptosis in MM cells by activating the Caspase-dependent signaling pathway, markedly suppressing the proliferation of CD138-CD34-MM CSCs, and exhibits favorable anti-tumor effects in both in vitro and in vivo experiments [94].

Specific ligands can be utilized on PNPs to implement targeted strategies, based on research on MM-related expression molecules. Researchers have documented the development of a drug carrier directed at CD38—daratumumab immunopolymersomes loaded with vincristine sulfate (Dar-IPs-VCR). Experimental findings indicate that Dar with a density of 4.4, named Dar4.4-IPs-VCR, exhibits the most potent anti-tumor activity against CD38-positive LP-1 MM cells. Interestingly, in vivo experiments in mice demonstrated complete eradication of tumor cells by Dar4.4-IPs-VCR, with the longest survival rate and absence of any organ damage [95]. Therefore, Dar-IPs-VCR holds promise as a safe and effective drug delivery system for MM treatment, with potential applicability to other CD38-overexpressing malignancies.

The reticuloendothelial system’s unresponsiveness or nonspecific clearance of nanoparticle ligands has hindered the advancement of nanomedicine [96]. Inspired by the characteristic of MM cells of homing to the bone marrow, QU et al. developed a biomimetic nanocarrier emulating MM by loading BTZ onto nanoparticles comprising poly(ε-caprolactone)-poly(ethylene glycol)-poly(ε-caprolactone) and enveloping them with MM cell membranes [97]. Upon intravenous injection, these nanoparticles utilize homing to the bone marrow, facilitated by surface molecules like CD44 [98] and CD147 [99,100] on MM cells, to access the BM cavity. Subsequently, they bind to tumor cells via homologous targeting proteins, impeding tumor growth. Moreover, research indicates that these nanoparticles can elude phagocytosis by the mononuclear phagocyte system, thereby extending circulation time. Hence, biomimetic delivery strategies may herald a novel approach to targeted drug delivery.

Polymers are ideal carriers in gene therapy. Small interfering RNA(siRNA) therapy silences pathogenic genes, potentially treating diseases like MM. BM endothelial cells expressing cyclophilin A and E-selectin play crucial roles in MM recurrence and drug resistance. Lipid-polymer nanocarriers targeted cyclophilin A [101] or co-silence E-selectin and cyclophilin A [102] in MM mouse xenograft models using siRNA. Compared with alone or BTZ, this prevented MM cell dissemination, extending mouse survival. MiR-34a induced by anticancer factor P53 is a promising MM therapy [103,104,105]. Chitosan/PLGA nanoplexes protect miR-34a from degradation, enabling gene therapy, with significant in vivo and in vitro anti-tumor effects, and improved survival rates [106].

In the direction of immunotherapy, the technology for encapsulating BCMA targeting peptides on the surface of nanoparticles is rapidly advancing. Due to the unique properties of PNP, installing poly(lactic-co-glycolic acid) (PLGA) with BCMA72-80 has increased the uptake of peptides by antigen-presenting cells compared to lipid-based delivery systems and induced BCMA-specific cytotoxic T lymphocytes with higher anti-tumor activity, providing direction for the development of PLGA-based MM therapeutic drugs [81]. Recently, BCMA-BTZ-NPs developed by DUTTA D. et al. have shown better inhibition of proteasome activity, induction of mitochondrial apoptosis, and reduced BTZ resistance, displaying high specificity and cytotoxicity against MM cells [107]. Liu, Zhaoyun et al. innovatively demonstrated the enhanced immunogenic cell death therapy of nanomedicine, confirming that loading aggregation-induced emission photosensitizers into nanoparticles of bovine serum albumin (BSA/TPA-Erdn) can activate T cells, reverse T cell senescence, and recruit more functional T cells into MM tumors, thereby restoring the MM microenvironment and finding new directions for MM treatment [108]. We can predict that immunotherapy based on nanomedicine delivery systems will become an important part of MM treatment.

### 5.4. Inorganic Nanoparticles

Inorganic nanoparticles are three-dimensional arrangement structures formed by the connection of atomic crystals between inorganic salt particles, capable of encapsulating both hydrophilic and hydrophobic drug molecules [66]. Compared with organic delivery carriers, they possess unique physical, electrical, magnetic, and optical properties [109]. They can be designed into specific sizes, structures, and shapes for drug or nucleic acid delivery and imaging purposes.

Metal nanoparticles like gold, silver, copper, zinc, and their oxides offer distinct advantages in drug delivery, while the majority of investigations are presently confined to in vitro studies. For example, the radioactive nano-complex ^131^I-Isatuximab/AgNPs, utilizing silver as a carrier and labeling with ^131^I, for transporting the anticancer drug isatuximab. It demonstrated markedly enhanced anti-proliferative and apoptotic effects on MM cells in comparison to individual components or their combinations in vitro [110]. Fe_3_O_4_ magnetic nanoparticles have gained widespread usage in biomedicine, encompassing drug delivery, cell labeling, magnetic resonance imaging, induction of enhanced ferroptosis, and immunotherapy [111,112,113,114]. Researchers have employed water-insoluble natural anticancer drug caffeic acid phenethyl ester (CAPE) and magnetic-resonance-imaging-trackable superparamagnetic iron oxide nanoparticles (IONPs) to fabricate RGD-IONP/CAPE, which specifically targets MM cells overexpressing integrin αvβ3 via receptor-mediated endocytosis. This formulation can fully harness the apoptotic effect induced by CAPE on MM cells (RPMI8226), mediated by caspase-9, enhancing biocompatibility and significantly inhibiting the growth of MM cells in vitro [112]. Nonetheless, this study is confined to in vitro settings, and further research is needed to ascertain the therapeutic efficacy of MM in the intricate tumor microenvironment in vivo. Furthermore, Fe_3_O_4_ magnetic nanoparticles loaded with paclitaxel [113] and loaded with BTZ and gambogic acid [114], respectively, have been demonstrated to ameliorate the inadequate water solubility of paclitaxel [66] and the high dose resistance and adverse reactions of BTZ [115,116] in both, underscoring their promising potential for targeted therapy of MM in in vitro and in vivo studies. Additionally, superparamagnetic iron oxide nanoparticles can be employed to localize T cells at target sites through magnetic manipulation without compromising their functionality, thus providing promise in aiding the prevalent immunotherapy in MM treatment [117].

Meanwhile, numerous metals and oxides exhibit intrinsic anticancer properties. Zinc oxide nanoparticles demonstrate both antibacterial and anti-tumor properties. In vitro, they significantly elevate reactive oxygen species production in RPMI8226 cells, upregulate the expression of Apaf-1, Atg5, Atg12, Becn1, Cyt-C, Caspase-3, and Caspase-9, reduce ATP levels, induce G2/M cell cycle arrest, and initiate autophagy signaling pathways, thereby exerting cytotoxic effects on MM cells [118,119]. They possess the potential for direct application as therapeutic agents in MM treatment. Gold nanoparticles, available in diverse forms, can impede the proliferation of MM cell lines by upregulating cell cycle protein-dependent kinase inhibitors p21 and p27, resulting in G1 phase cell cycle arrest [120,121].

## 6. Prevention and Management of Adverse Events

In the context of novel therapeutic interventions for MM, a majority of patients have reported experiencing adverse events related to treatment. Distinguishing whether these complications arise from the pharmacological intervention or the natural progression of the disease is challenging, particularly as MM patients typically undergo combination therapy to optimize treatment efficacy. Consequently, attributing specific side effects to an individual drug becomes problematic. According to a meta-analysis [122], ADCs carrying various drugs can elicit distinct adverse reactions. For instance, ADCs with MMAE have been consistently associated with Grade 3/4 anemia, neutropenia, and peripheral neuropathy, while those with DM1 are linked to thrombocytopenia and hepatotoxicity, and ADCs with MMAF demonstrate ocular toxicity. Subsequently, we provide a comprehensive overview of the prevalent adverse reactions encountered with new MM DDSs, alongside strategies for their management and prevention.

### 6.1. Hematologic Toxicities

Hematologic toxicities are the most prevalent adverse reactions associated with novel anti-MM drugs, manifesting as neutropenia, anemia, thrombocytopenia, and pancytopenia. It remains challenging to determine whether the reduction in blood cell counts is a consequence of the disease’s progression or an effect of the novel treatments.

In multiple clinical trials of belantamab mafodotin, significant hematologic toxicity has been consistently observed. For instance, the DREAMM-3 clinical trial highlighted that thrombocytopenia (23%) and anemia (16%) were the most frequent Grade 3–4 adverse events in the treatment cohort [29]. Similarly, melflufen was predominantly associated with hematologic toxicity, with the phase 3 OCEAN study reporting high incidences of Grade 3 or 4 thrombocytopenia (63%), neutropenia (54%), and anemia (43%) [53].

These adverse events are generally manageable through interventions such as dose adjustments, scheduling delays, growth factor administration, blood cell transfusions, and supportive care. Chemotherapy-induced anemia is generally normocytic and normochromic. Red blood cell transfusions can be beneficial for patients needing an immediate improvement of anemic conditions. Erythropoiesis-stimulating agents such as erythropoietin (Epo)-α and β and darbepoetin have been shown to elevate hemoglobin levels in MM patients, reducing transfusion requirements and enhancing quality of life [123]. Neutropenia increases the risk of severe bacterial infections, leading to higher hospitalization and mortality rates. Granulocyte colony-stimulating factors, introduced clinically in the 1990s, continue to be the standard treatment for chemotherapy-induced neutropenia [124]. Prophylactic quinolone antibiotics, preferably levofloxacin, are recommended for patients with prolonged neutropenia (absolute neutrophil count < 1000/µL) exceeding 7 days [125]. In cases of thrombocytopenia, prophylactic transfusions of platelet concentrates are advised when platelet counts fall below 10 × 10^−9^/L to prevent bleeding [126]. Ongoing research into platelet production drugs has identified recombinant interleukin-11 as the only FDA-approved drug for chemotherapy-induced thrombocytopenia. Additionally, second-generation thrombopoietin receptor agonists, such as romiplostim and eltrombopag, have been proven effective [127].

### 6.2. Ocular Disorders

The majority of patients undergoing treatment with belantamab mafodotin exhibit corneal changes, observable as superficial punctate keratopathy and microcystic epithelial changes (MECs) under slit-lamp microscopy [128]. Superficial punctate keratopathy encompasses non-inflammatory alterations in the cornea’s outer layer, while MEC consists of cyst-like microdeposits. Affected individuals may experience symptoms such as blurred vision, a decline in best corrected visual acuity, keratitis, and dry eye syndrome. However, a direct correlation between these corneal changes and vision deterioration has not been established. No permanent visual alterations or losses have been reported in patients treated with belantamab mafodotin to date, with most corneal changes being reversible or resolving post-treatment-cessation [129]. The underlying mechanisms of corneal changes remain elusive, but they are hypothesized to be an off-target effect of belamaf-induced apoptosis in corneal epithelial cells. Preclinical studies have substantiated that corneal cells do not express BCMA. Post-cellular destruction by belamaf, a significant release of sBCMA into the bloodstream occurs, followed by an increase in tear sBCMA levels. This leads to the formation of sBCMA-belamaf complexes in tears, which are internalized by corneal epithelial cells via receptor-ligand-independent pinocytosis, culminating in corneal changes [130].

The KVA scale for belamaf-related corneal events categorizes these into four grades [128]. Grade 1 is identified by a sparse presence of MECs, predominantly peripheral, with a one-line reduction in Snellen Visual Acuity from the baseline, warranting continuation of the current dosage. Grade 2 presents with moderate MEC density, primarily paracentral, with a two- to three-line decrease in BCVA from baseline, necessitating treatment suspension until improvement to Grade 1, followed by a reduced dose resumption. Grade 3 involves a high density of MECs, centered in the cornea, with a BCVA reduction exceeding three lines from baseline, managed similarly to Grade 2. Grade 4 features corneal epithelial defects, including ulcers, requiring treatment suspension until improvement, with discontinuation considered after a benefit–risk assessment. No specific prophylactic or therapeutic measures for belamaf-related corneal events exist. However, dose adjustment of belamaf, the use of preservative-free lubricating eye drops, and minimizing contact lens wear are recommended. Corticosteroid eye drops have proven ineffective against MEC prevention [28].

### 6.3. Peripheral Neuropathy

Peripheral neuropathy is a frequent adverse effect associated with microtubule-targeting agents in clinical trials. A meta-analysis on the clinical toxicity of ADCs indicated a heightened risk of peripheral neuropathy with ADCs containing microtubule inhibitors, especially MMAE [122]. The ADC DFRF4539A, targeting FcRH5 and carrying MMAE, demonstrated peripheral neuropathy in 21% of patients (8 out of 38) in its initial human trial [40]. Similarly, IMGN901, an ADC with DM1, observed a 51% incidence of drug-related peripheral neuropathy (19 out of 37 patients), which was the leading cause of discontinuation due to treatment-related adverse events [131].

The neuropathy induced by microtubule-inhibitor-based ADCs typically manifests as sensory neuropathy, characterized by pain, numbness, paresthesia, hypoesthesia, and hyperesthesia, with motor neuropathy being infrequent [132]. The proposed mechanism involves damage to axonal terminals, DRG neuronal cells, and Schwann cells. MMAE disrupts the microtubule network, inhibits mitosis, and blocks axonal transport when internalized by peripheral nerve cells, resulting in severe neuropathy [133]. Management strategies for ADC-induced neuropathy include dose adjustment, treatment suspension, or permanent cessation based on adverse reaction severity. Research suggests the potential benefits of pharmacological treatments like mecobalamin, acetyl-L-carnitine, amifostine, and duloxetine, and non-pharmacological interventions such as acupuncture, cryotherapy, and compression therapy in mitigating neuropathy symptoms [134].

### 6.4. Infusion-Related Reactions

Infusion-related reactions (IRRs) are frequently encountered in ADCs for MM treatment. For instance, the initial phase 1 trial of TAK-573 reported IRRs in 33% of participants [39], and CX-2029 treatment saw IRRs as the most common TEAE at 87%, predominantly grades 1 and 2 [45].

IRRs, adverse responses to infused drugs or biologicals, are generally dose- and pharmacology-independent, unpredictable, and reversible upon infusion cessation [135]. They may be immune-mediated, such as allergic reactions, or non-immune-mediated, including cytokine release syndrome, idiosyncratic reactions, and intolerance [136]. Common IRR manifestations involve skin and mucosal symptoms (itching, urticaria, flushing), respiratory issues (wheezing, breathlessness), circulatory effects (hypotension), and gastrointestinal symptoms [137]. Anticancer therapy protocols that may elicit IRRs necessitate comprehensive patient risk assessment, progressive infusion rates, and recommended medication with corticosteroids (e.g., dexamethasone), antihistamines (e.g., diphenhydramine), and antipyretics [138].

Adverse reactions linked to drug-conjugate system therapies are predominantly attributed to the payload drugs and off-target effects. These new drugs necessitate vigilant monitoring and prompt intervention by healthcare professionals, including dose modification, treatment discontinuation, and supportive care. Optimizing ADC design to substantially reduce off-target effects represents the most hopeful strategy for mitigating adverse events.

## 7. Discussion

For decades, with an improved understanding of MM surface targets and the application of various new materials, we have designed and developed a wide range of DDS to enhance MM treatment. Since the concept of ADC was proposed in 1960, the success of ADCs has marked the beginning of the era of ‘coupling everything’. Several ADC drugs have completed Phase I clinical trials for MM, showcasing promising prospects in the ADC drug development field. However, ADCs encounter challenges, including limited cellular penetration due to their size, potential antibody-induced immunogenicity causing adverse reactions, and high production costs. Recent clinical trials of novel ADCs were halted due to low efficacy and substantial toxic side effects [19]. By the end of 2022, Blenrep, the sole FDA-approved ADC drug for MM, was withdrawn from the U.S. market as it failed to demonstrate significant benefits in progression-free survival in the Phase III DREAMM-3 (DRiving Excellence in Approachs to Multiple Myeloma-3) studies. These findings underscore the need for more rigorous clinical trials to validate the application of ADC drugs in MM treatment. Combining ADCs with currently effective anti-MM drugs is an effective approach that enhances therapeutic efficacy while reducing toxic side effects. As demonstrated by the results of the DREAMM-7 and DREAMM-8 trials, the combination of belantamab mafodotin with other anti-MM drugs has significant therapeutic effects, injecting new vitality into the application of belantamab mafodotin.

PDCs represent a promising field rooted in ADC research, addressing ADC limitations. With lower molecular weight, PDCs penetrate tissues effectively, exhibit low immunogenicity, and enzymatically produce safe metabolites. PDCs offer simple synthesis and enhanced peptide targeting via chemical modification. Recent PDC research has focused on combination therapy, like melflufen, for relapsed or refractory MM in adults. However, the failure of Phase III clinical trials has led to its withdrawal from the clinical market. The unresolved issues of poor circulation stability and rapid renal clearance raise doubts about whether PDC is just a flash in the pan.

ApDCs represent a modification established on the foundation of ADCs and are currently in the early stages of development, with only a limited number of ApDCs advancing to clinical trials. In contrast to antibodies, aptamers offer several advantages, including simplicity in synthesis, ease of chemical modification, exceptional stability, and cost-effectiveness. Nevertheless, a series of in vivo experiments have revealed several concerns, including the drug’s rapid renal clearance and the inherent conflict between drug dosage and target specificity.

Nanoparticle DDSs are currently a hot research topic. These systems use nanoscale materials for drug encapsulation, allowing precise control of drug release. They offer multifunctionality, high drug capacity, and extended retention times [139], improving traditional drug limitations, reducing side effects, and enhancing therapy. However, challenges remain. Insufficient preclinical models have led to shortcomings in targeting efficiency and the ability to observe potential long-term toxicity [140]. While liposomes are currently the most well-known and widely used carriers in nanomedicine, they may face difficulties in achieving substantial and stable drug loads due to volume and water solubility limitations, potentially leading to low drug delivery system efficiency and poor cost-effectiveness [141]. Moreover, the 1–100 nm size of nanoparticles results in high surface energy, the spherical shape of nanoparticles leads to rolling friction, and attaching antibodies to the particle surface for targeted therapy modifies surface characteristics, while various other factors contribute to nanoparticle aggregation. This phenomenon is particularly pronounced with inorganic nanoparticles, exacerbating drug instability, escalating drug preparation costs, and increasing the complexity of research and development efforts.

This review has several limitations that should be noted. Firstly, the discovery of therapeutic targets forms the foundation for the development of targeted DDSs. The primary objective of this paper is to introduce current advancements in DDSs targeting MM, which results in an incomplete introduction of MM targets. For instance, monoclonal antibody elotuzumab targeting SLAMF7 (also known as CD319 or CS1) is not discussed. Secondly, research on nanodrug delivery systems for MM is predominantly focused on cellular and animal experimentation stages, with very few drugs progressing to clinical research. Consequently, there is a lack of evaluation on the long-term efficacy and safety of nanomedicine in the treatment of MM, as well as research on its clinical application and dissemination.

To address the aforementioned issues, a deeper understanding of the pathophysiological changes in MM is required. This involves identifying more precise targets and developing various ligands with higher specificity, such as antibodies, peptides, aptamers, and other ligands, for instance, introducing non-natural nucleic acids to enrich aptamer libraries, developing efficient nucleic acid amplifying enzymes capable of recognizing non-natural nucleic acids, and searching for or developing ApDCs with both high payload and high targeting capabilities. Installing two targeting ligands on delivery systems or using bispecific ligands may also enhance the targeting specificity for MM, thereby reducing toxicity to other sites. Improving MM diagnostic and screening technologies to promptly identify changes in MM-related antigens and targets will contribute to the advancement of various targeted delivery systems. Additionally, more patient-relevant animal models need to be established to provide a solid foundation for clinical trials. As novel targeted DDSs, comprehensive long-term toxicity data for these drugs are still insufficient, and continued monitoring is required. In summary, the rapid development of various targeted DDSs holds promising prospects for targeted therapy in MM.

## Figures and Tables

**Table 1:** Antibody–drug conjugates useful in multiple myeloma.

ADC	Target	Payload	Linker	Mechanism of Action	Adverse Event	Clinical Status
TAK-169	CD38	Shiga-like toxin-A subunit (SLTA)	/	Enzymatic inactivation of cell ribosomes [38]	Myocarditis	Phase 1 (NCT04017130, terminated)
TAK-573 (Modakafusp Alfa)	CD38	attenuated interferon alpha-2b (IFNα2b)	/	Direct anti-proliferative effects, and induce immune cell activation [39]	Infusion-related reactions (IRR), Hematologic toxicity	Phase 1/2 (NCT05590377, active, not recruiting)
DFRF4539A	FcRH5	MMAE	protease-labile linker (MC-VC-PABC)	Disruption of microtubule networks [40]	Anemia, fatigue, peripheral neuropathy	Phase 1 (NCT01432353, completed)
Indatuximab ravtansine (BT062)	CD138	DM4	disulphide bonds (SPDB)	Disruption of microtubule networks [41]	Diarrhea and fatigue	Phase 1/2a (NCT01638936, completed)
Milatuzumab doxorubicin (hLL1-DOX)	CD74	doxorubicin	acid-labile linker (hydrazone)	Preventing DNA replication and increasing double-strand breaks [42]	/	Phase 1/2 (NCT01101594, terminated)
Lorvotuzumab mertansine (IMGN901)	CD56	DM1	Disulphide bonds (SPP)	Disruption of microtubule networks [43]	peripheral neuropathy	Phase 1 (NCT00991562, completed)
FOR46	CD46	MMAE	protease-cleavable linker (mcvcpab)	Disruption of microtubule networks [44]	/	Phase 1 (NCT03650491, completed)
CX-2029	CD71	MMAE	protease-cleavable linker	Disruption of microtubule networks [45]	Hematologic toxicity, IRR	Preclinical

## Data Availability

Data sharing is not applicable.
